# P2X_4_ deficiency reduces atherosclerosis and plaque inflammation in mice

**DOI:** 10.1038/s41598-022-06706-6

**Published:** 2022-02-18

**Authors:** Alexander Peikert, Sebastian König, Dymphie Suchanek, Karlos Rofa, Ibrahim Schäfer, Daniel Dimanski, Lorenz Karnbrock, Kseniya Bulatova, Juliane Engelmann, Natalie Hoppe, Carolin Wadle, Timo Heidt, Philipp Albrecht, Sunaina von Garlen, Carmen Härdtner, Ingo Hilgendorf, Dennis Wolf, Constantin von zur Mühlen, Christoph Bode, Andreas Zirlik, Daniel Duerschmied, Julian Merz, Peter Stachon

**Affiliations:** 1 Department of Cardiology and Angiology I, University Heart Center Freiburg, Medical Faculty, University of Freiburg, Hugstetter Straße 55, 79106 Freiburg, Germany; 2 Department of Cardiology, Medical University of Graz, Graz, Austria; 3 First Department of Medicine (Cardiology), Medical Faculty Mannheim, University Medical Centre Mannheim, Mannheim, Germany

**Keywords:** Cardiovascular biology, Experimental models of disease

## Abstract

Extracellular adenosine-5′-triphosphate (ATP) acts as an import signaling molecule mediating inflammation via purinergic P2 receptors. ATP binds to the purinergic receptor P2X_4_ and promotes inflammation via increased expression of pro-inflammatory cytokines. Because of the central role of inflammation, we assumed a functional contribution of the ATP-P2X_4_-axis in atherosclerosis. Expression of P2X_4_ was increased in atherosclerotic aortic arches from low-density lipoprotein receptor-deficient mice being fed a high cholesterol diet as assessed by real-time polymerase chain reaction and immunohistochemistry. To investigate the functional role of P2X_4_ in atherosclerosis, P2X_4_-deficient mice were crossed with low-density lipoprotein receptor-deficient mice and fed high cholesterol diet. After 16 weeks, P2X_4_-deficient mice developed smaller atherosclerotic lesions compared to P2X_4_-competent mice. Furthermore, intravital microscopy showed reduced ATP-induced leukocyte rolling at the vessel wall in P2X_4_-deficient mice. Mechanistically, we found a reduced RNA expression of CC chemokine ligand 2 (CCL-2), C-X-C motif chemokine-1 (CXCL-1), C-X-C motif chemokine-2 (CXCL-2), Interleukin-6 (IL-6) and tumor necrosis factor α (TNFα) as well as a decreased nucleotide-binding oligomerization domain-like receptor protein 3 (NLRP3)-inflammasome priming in atherosclerotic plaques from P2X_4_-deficient mice. Moreover, bone marrow derived macrophages isolated from P2X_4_-deficient mice revealed a reduced ATP-mediated release of CCL-2, CC chemokine ligand 5 (CCL-5), Interleukin-1β (IL-1β) and IL-6. Additionally, P2X_4_-deficient mice shared a lower proportion of pro-inflammatory Ly6C^high^ monocytes and a higher proportion of anti-inflammatory Ly6C^low^ monocytes, and expressend less endothelial VCAM-1. Finally, increased P2X_4_ expression in human atherosclerotic lesions from carotid endarterectomy was found, indicating the importance of potential implementations of this study’s findings for human atherosclerosis. Collectively, P2X_4_ deficiency reduced experimental atherosclerosis, plaque inflammation and inflammasome priming, pointing to P2X_4_ as a potential therapeutic target in the fight against atherosclerosis.

## Introduction

Cardiovascular diseases and their complications such as myocardial infarctions, strokes and heart failure represent the leading causes of mortality worldwide^1^. Chronic inflammation is a key process linking traditional cardiovascular and metabolic risk factors with the clinical onset and acceleration of cardiovascular disease^2–5^. Despite therapeutic progress in anti-inflammatory treatments in cardiovascular disease, clinical trials raised concerns about increasing infections under recently investigated therapies with canakinumab or colchicine^6,7^. To surmount these complications, the development of more targeted alternative strategies that address specific mechanisms of cardiovascular inflammation is of paramount importance.

Nucleotides such as adenosine-5′-triphosphate (ATP) play a crucial role as cellular energy carrier. During inflammatory activation, invoked by ischemia, cellular stress or cell death, nucleotides are released into the extracellular space^8^. Extracellular nucleotides act as important signaling molecules mediating inflammation via the purinergic P2 receptors, which in turn are divided into G-protein-coupled P2Y receptors and ligand-gated ion channel P2X receptors^9^. Purinergic P2 receptors are involved in the pathogenesis of a multitude of chronic inflammatory diseases like asthma, chronic obstructive pulmonary disease, inflammatory bowel disease or graft versus host disease^10–12^. Simultaneously, purinergic receptors perform an important function in the pathophysiology of atherosclerosis. Recent studies showed that the metabotropic receptors P2Y_1_, P2Y_2_, P2Y_6,_ and the ligand-gated receptor P2X_7_ lead to experimental atherosclerosis by mediating leukocyte recruitment to the vessel wall and caused vascular inflammation by promoting adhesion molecule expression, cytokine expression as well as nucleotide-binding oligomerization domain-like receptor protein 3 (NLRP3)-inflammasome activation^13–17^.

The purinergic receptor P2X_4_ is a ligand-gated ion channel responsive to ATP and permeable to Na^+^, K^+^ as well as Ca^2+^ ions. It is the most sensitive purinergic receptor at nanomolar ATP concentrations and at the same time the most expressed P2X receptor subtype in the heart^18,19^. The distribution of P2X_4_ extends over a variety of stromal cell types including endothelial cells, smooth muscle cells, cardiomyocytes, epithelial cells, fibroblasts, peripheral and central neurons as well as leukocytes like dendritic cells, eosinophils and macrophages. While P2X_4_ is primarily stored intracellularly in lysosomes, the expression of P2X_4_ on the cell surface is extremely sensitive and particularly upregulated by stimuli such as ischemia or inflammation^20,21^. Hence, P2X_4_ is known to mediate inflammation in several disease models like kidney injury, chronic lung disease, rheumatoid arthritis and graft-versus-host disease. Activation of P2X_4_ in response to high extracellular ATP levels evokes the release of cytokines like Interleukin 6 (IL-6) and tumor necrosis factor α (TNFα), promoting NLRP3-inflammasome signaling as well as activation of endothelial cells^22–24^.

Based on this background information, the aim of this study is to investigate the role of P2X_4_ in experimental atherosclerosis and vascular inflammation.

## Materials and methods

### Mice

All methods are reported according to the ARRIVE guidelines. P2X_4_-deficient mice on homozygous C57Bl/6 background (P2rx4^*tm1Rass*^) were kindly provided by Prof. Dr. Idzko (Medical University of Vienna). LDL-receptor knockout mice on C57Bl/6 background (Ldlr^*tm1Her*^) were purchased from Jackson Laboratories (Bar Harbor, Maine, USA). By crossing P2X_4_-deficient mice with LDLR-deficient animals, P2X_4_^−/−^/LDLR^−/−^ double knockout animals were generated. Animals were kept under specifically pathogen-free (SPF) conditions at the animal facility of the University of Freiburg. All procedures were approved by the governmental animal care committee (Regierungspraesidium Freiburg, Germany, 35-9185.81/G-17/172) and conformed to the guidelines from the EU directive 2010/63 EU of the European Parliament.

### In vivo atherosclerosis study

6 weeks old, male, P2X_4_^−/−^/LDLR^−/−^ mice (n = 24) and P2X_4_^+/+^/LDLR^−/−^ mice (n = 15) were fed high cholesterol diet (sniff EF R/M acc. D12108 mod., ssniff Spezialdiäten GmbH, Soest, Germany) for 16 weeks. After 16 weeks of diet, mice were harvested as previously described^15^.

### Histology

From the aortic samples of the atherosclerosis study, sections were prepared for histological and immunohistochemical preparations according to the protocol described before^15^. Stainings with Oil-red-O, anti-Mac-3, Sirius red and anti-α-actin were performed as described previously^15^. Anti-CD4 staining was realized using a CD4 immunofluorescence kit (Sigma-Aldrich, St. Luis, MO, USA) according to the manufacturer’s protocol. Plaque size was determined in sections of the aortic root, arch and abdominal aorta by analyzing total wall area, intimal lesion area, and medial area. Necrotic core size was analyzed by measuring acellular intima lesion areas in sections of the aortic root as previously described^25^. For the analysis of plaque composition, the percentage of positively stained area was quantified for lipids (Oil-Red-O), macrophages (anti-mouse Mac-3), collagen (Sirius red), smooth muscle cells (anti-α-actin), and CD4+ cells (anti-CD4) within the intimal lesion area.

Staining of active caspase-1 in aortic roots was made as described previously using a FLICA-fmk kit (Immunohistchemistry Technologies)^17^. The percentage of FLICA positive area was quantified.

To determine the expression of P2X_4_ in aortic roots, we performed a P2X_4_ staining using a P2X_4_ antibody (United States Biological, Salem, MA, USA).

For 3-colour-immunofluorescence, stainings for cell nuclei (DAPI) and endothelial cells (anti-CD31) were made as previously described^14,15^. Immunofluorescence staining of P2X_4_ was performed using a P2X_4_ immunofluorescence antibody according to the manufacturer’s protocol (Thermo Fisher Scientific, Waltham, MA, USA).

For analysis of endothelial expression of VCAM-1 and ICAM-1, 3-colour-immunofluorescence with additional stainings for cell nuclei (DAPI) and endothelial cells (anti-CD31) were prepared as described previously^14,15^. The area of the CD31/VCAM-1 and ICAM-1 positive signals was measured.

All analyses were performed by blinded investigators using Image Pro software (Media Cybernetics, Rockville, MD, USA) as previously described^26^.

### Intravital microscopy

P2X_4-_competent and P2X_4_-deficient mice were stimulated intraperitoneally with 500 µL of 50 μM ATP-γ-S (Sigma-Aldrich, St. Luis, MO, USA) diluted in PBS or 500 μL PBS. 2 h later, mice were anesthetized by a intraperitoneal injection of 80% 100 mg/mL ketamine hydrochloride (Freiburg Inresa Arzneimittel, Freiburg, Germany) and 20% 20 mg/mL xylazin (Rompun 2%, Bayer Vital GmbH, Leverkusen, Germany). Leukocytes were stained by retro-orbital injection of 60 µL rhodamin 6G (1 mg/mL). Animal preparation and exposure of mesenteric vessels was conducted as described previously^15^. Leukocyte characteristics were observed using an intravital fluorescence microscope (AxioScope Vario, Carl Zeiss Inc., Oberkochen, Germany). Rolling and adhesion of leukocytes was quantified with Axiovision Rel. 4.6 Software (Carl Zeiss Inc., Oberkochen, Germany) by a blinded investigator.

### Quantitative reverse transcript polymerase chain reaction

In order to determine mRNA expression levels in aortic lesions of the atherosclerosis study, RNA expression in aortic arches was analyzed. RNA extraction from aortic arches as well as purification and reverse transcription of isolated RNA were carried out as specified before^15,17^. To conduct real-time polymerase chain reaction, fluorochrome-tagged TaqMan primers were used (Thermo Fisher Scientific, Waltham, MA, USA). Detection and amplification of samples were carried out using a CFX96 TaqMan system (Biorad Laboratories, Hercules, CA, USA). Analysis of semiquantitative mRNA expression was performed by ddCt method, results were referred to β-Actin as the housekeeping gene.

### Cell isolation and cell culture

In order to perform bone marrow cell analysis and further experiments with bone marrow-derived macrophages (BMDMs), hematopoietic progenitor cells were isolated from the bone marrow of 8-week-old P2X_4_-competent and P2X_4_-deficient mice as previously described^17^. The isolated progenitor cells were resuspended in Bone Marrow Medium (Thermo Fisher Scientific, Waltham, MA, USA)) at 10^6^ cells/mL. For analysis of common myeloid progenitor cells, the progenitor cells were phenotyped by fluorescence-activated cell sorting cytometry based on CD45^+^, Lin^−^ (CD3^−^, CD19^−^, NK1.1^−^, Gr-1^−^, CD 11b^−^, and Il7Ra^−^), cKit^+^, and Sca-1^−^ cell surface markers as previously described^17,27^.

To obtain BMDMs, 10^6^ progenitor cells per well were differentiated by the addition of 3 µg/mL M-CSF for 7 days. Fully differentiated bone marrow-derived macrophages (BMDMs) were first stimulated with 100 ng/mL LPS (*E. coli*, Sigma Aldrich) for 4 h, followed by stimulation with either 100 µM or 5 mM ATP (Sigma Aldrich) for 1 h. Supernatant samples were collected and snap-frozen in liquid nitrogen for further analysis.

Cytometric analysis of bone marrow cell subsets and cytokine concentrations were performed using a BD FACSCanto™ II (Becton Dickinson & Company), and offline analysis was done using FlowJo™ Software (Version 10.7.1. Becton, Dickinson and Company).

### Inflammatory cytokine quantification

Plasma from euthanized P2X_4_^−/−^/LDLR^−/−^ and LDLR^−/−^ male mice after 16 weeks of high-cholesterol diet as well as supernatants from bone marrow-derived macrophage cell cultures were analyzed using a multiplex assay utilizing fluorescence-encoded beads (LEGENDplex Multi-Analyte Flow Assay Kit, BioLegend, San Diego, CA, USA). The concentrations of interferon-β (IFN-β), interferon-γ (IFN- γ), Interleukin-1β (IL-1β), IL-6, Interleukin 10 (IL-10), Interleukin 12 (IL-12), CC chemokine ligand 2/monocyte chemotactic protein 1 (CCL-2/MCP-1), CC chemokine ligand 5 (CCL5), C-X-C motif chemokine-1 (CXCL-1), and TNF-α were determined using the multiplex fluorescence-encoded beads assay according to the manufacturer's protocol.

### Human atherosclerosis study

Human carotid arteries of patients with atherosclerotic carotid plaques were prepared after endarterectomy for histological sections. The carotid sections were stained against P2X_4_ (Thermo Fisher Scientific, Waltham, MA, USA). Expression of P2X_4_ was determined in non-diseased and atherosclerotic areas of the same carotid sections. Additionally, 3-colour-immunofluorescence stainings for P2X_4_, cell nuclei (DAPI) and endothelial cells (anti-CD31) were performed. Analysis was performed by blinded investigators using Image Pro software (Media Cybernetics, Rockville, MD, USA).

### Data analysis

Data of > 3 experiments were pooled and results are presented as mean ± SEM. Statistical analyses were performed using unpaired t-test for parametric data or Mann–Whitney-*U*-test for non-parametric data. Normal distribution of results prior to further analysis was verified by Shapiro–Wilk Test. A probability value ≤ 0.05 was considered as statistically significant.

### Ethics approval

This study was approved by the governmental Animal Care committee. All experiments conformed to the guidelines from the EU directive 2010/63 EU of the European Parliament.

## Results

### P2X_4_ expression is increased in murine atherosclerosis

**Figure 1:**
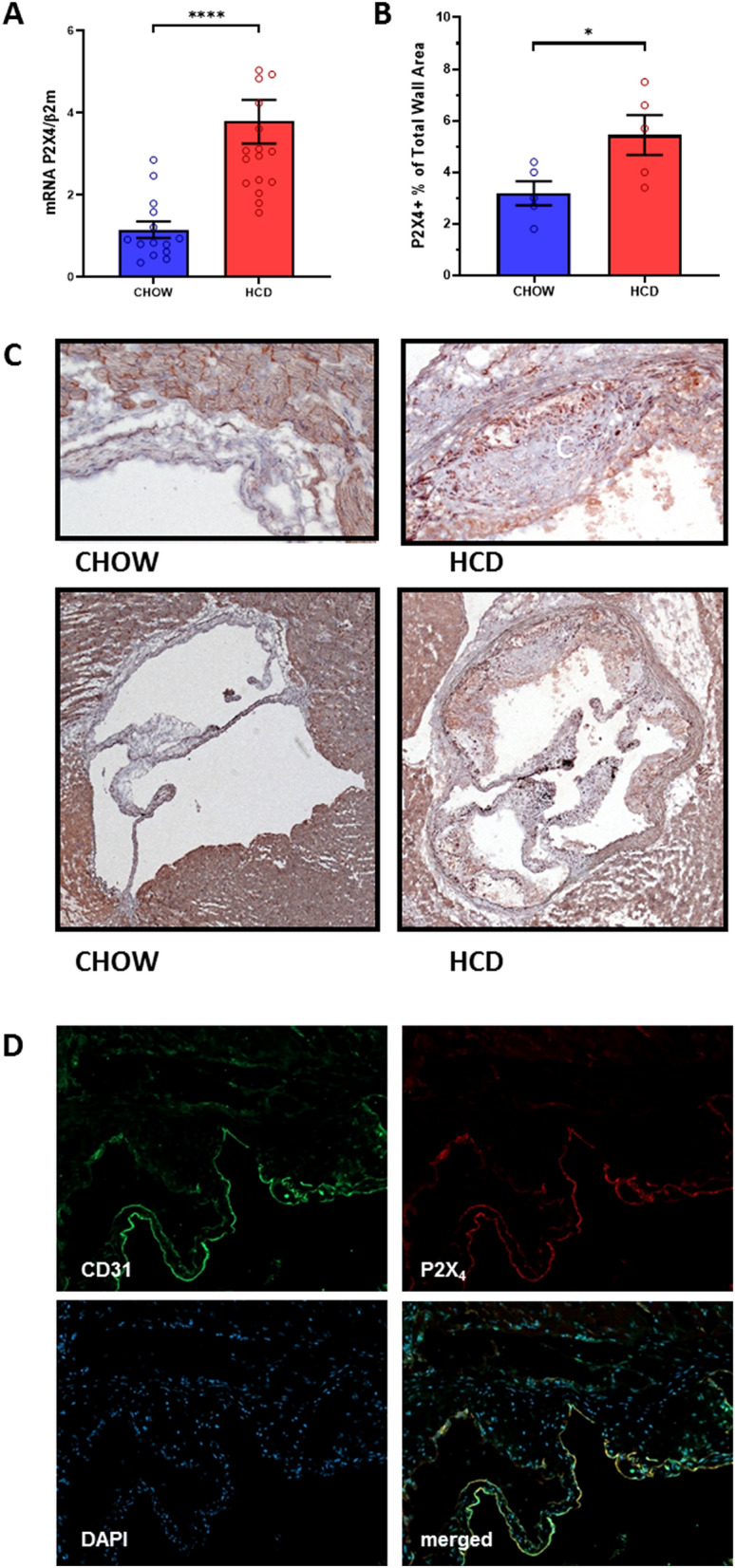
P2X_4_ is expressed murine atherosclerosis. LDL-R^−/−^ mice consumed either a high cholesterol diet (n = 16) or a chow diet (n = 14) for 16 weeks. After diet, RNA was isolated from aortic arches and P2X_4_ expression was assessed by quantitative polymerase chain reaction (**A**). Aortic roots (n = 10) were stained with anti-P2X4, and P2X_4_-positive staining area were quantified within the aortic sections (**B**, **C**). Distribution of P2X_4_ in atherosclerotic lesions from LDLR^−/−^ mice (n = 15) was analyzed by 3-colour immunofluorescence staining for cell nuclei (DAPI, blue), endothelial cells (CD 31, green) and P2X_4_ (red). Representative sections are shown in the merged images (**D**). Results are presented as mean ± SEM. Statistical significance was calculated using unpaired t-test (parametric data) or Mann–Whitney-*U*-test (non-parametric data). *p < 0.05; ****p < 0.0001.

In order to investigate whether P2X_4_ is present in atherosclerosis, we analyzed the expression of P2X_4_ in murine atherosclerotic plaques. Therefore, low-density lipoprotein receptor-deficient (LDLR^−/−^) mice were fed a high cholesterol diet for 16 weeks to evoke atherosclerosis (n = 16). As a control, low-density lipoprotein receptor-deficient (LDLR^−/−^) mice consumed chow diet (n = 14). Following the diet period, quantitative real-time polymerase chain reaction revealed a highly significant increase of P2X_4_ expression in aortic arches with atherosclerotic lesions (*p* < 0.0001, Fig. 1A), compared to non-atherosclerotic aortic tissue. Moreover, immunohistochemical analysis showed that atherosclerotic aortic roots (n = 5) showed significantly more pronounced P2X_4_-positive areas than sections from non-diseased aortas (n = 5) (*p* < 0.05, Fig. 1B,C). To evaluate the distribution of P2X_4_ within atherosclerotic lesions, we performed a 3-colour immunofluorescence staining for cell nuclei (DAPI), endothelial cells (CD 31) and P2X_4_ (n = 15). Accord to immunohistochemistry, P2X_4_ positive staining was particularly colocalized with CD31 positive endothelial cells (Fig. 1D).

### P2X_4_-deficiency reduces atherosclerosis

**Figure 2:**
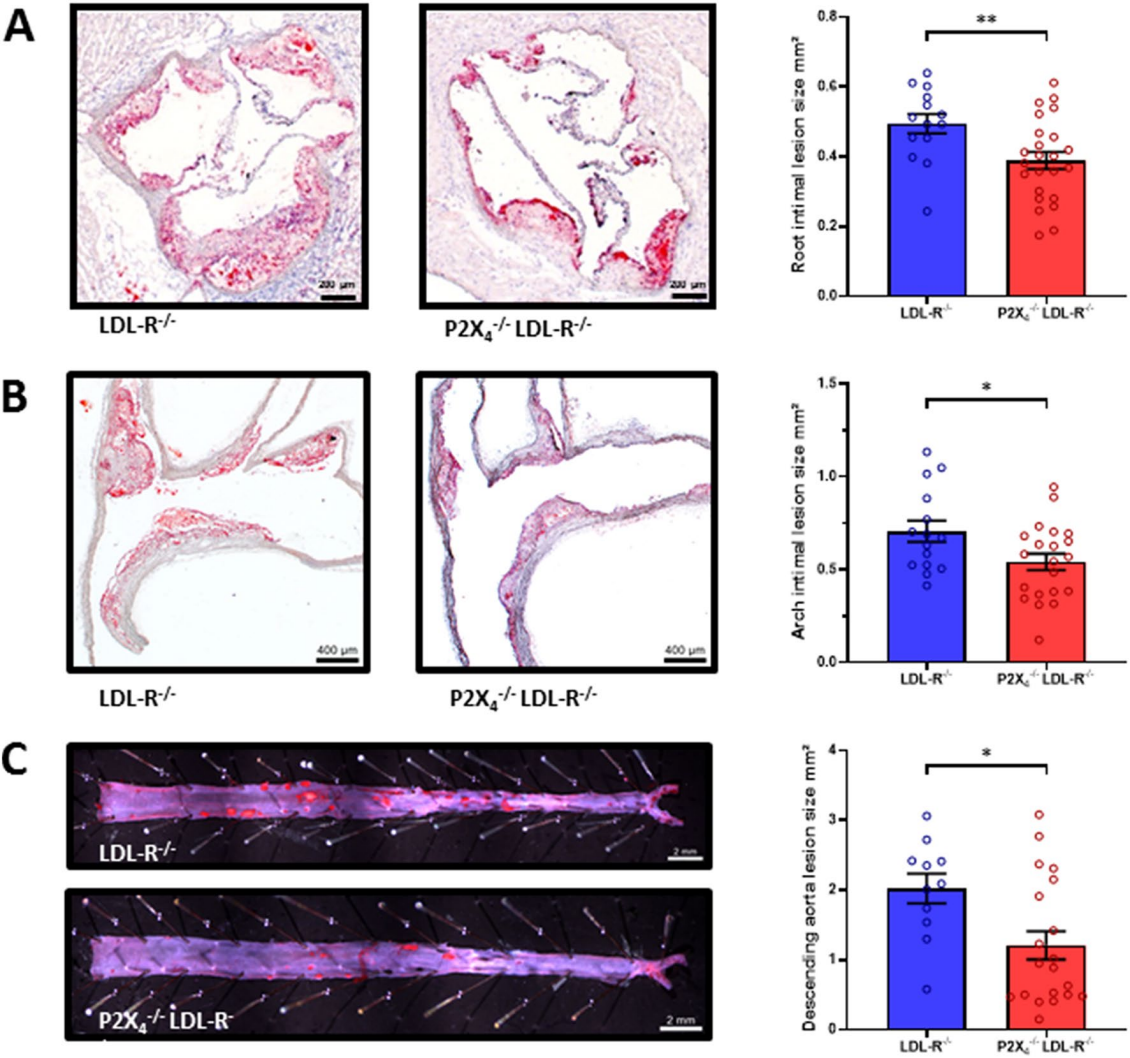
P2X_4_-deficiency reduces atherosclerosis. P2X_4_^−/−^ LDLR^−/−^ mice (n = 24) and P2X_4_^+/+^ LDLR^−/−^ mice (n = 14) were fed a high-cholesterol diet for 16 weeks. Atherosclerotic lesion size in aortic roots (**A**), aortic arches (P2X_4_^−/−^ LDLR^−/−^ n = 21, P2X_4_^+/+^ LDLR^−/−^ n = 15) **(B**) and abdominal aortas (P2X_4_^−/−^ LDLR^−/−^ n = 20, P2X_4_^+/+^ LDLR^−/−^ n = 11) (**C**) was determined by histochemistry, representative images are shown. Results are presented as mean ± SEM. Statistical significance was calculated using unpaired t-test (parametric) or Mann–Whitney*-U*-test (non-parametric data). *p < 0.05; **p < 0.01.

To elucidate the functional role of P2X_4_ in atherosclerosis, P2X_4_-deficient mice were crossed with LDLR^−/−^ mice and fed a HCD for 16 weeks. P2X_4_^+/+^ LDLR^−/−^ littermates were used as control. Following 16 weeks of HCD, intimal lesion size in aortic roots, aortic arches, and abdominal aortas was determined histochemically. P2X_4_^−/−^ LDLR^−/−^ mice developed significantly smaller atherosclerotic lesions in the aortic root (P2X_4_^+/+^ LDLR^−/−^: 0.49 ± 0.11mm^2^, n = 15; P2X_4_^−/−^ LDLR^−/−^: 0.39 ± 0.12 mm^2^, n = 24; *p* < 0.01; Fig. 2A) and the aortic arch (P2X_4_^+/+^ LDLR^−/−^: 0.71 ± 0.22mm^2^, n = 15; P2X_4_^−/−^ LDLR^−/−^: 0.54 ± 0.21mm^2^, n = 21; *p* < 0.05; Fig. 2B). Moreover, intimal lesion size in the abdominal aorta was significantly reduced in P2X_4_^−/−^ LDLR^−/−^ mice compared to control (P2X_4_^+/+^ LDLR^−/−^: 2.02 ± 0.7mm^2^, n = 11; P2X_4_^−/−^ LDLR^−/−^: 1.21 ± 0.9mm^2^, n = 20; *p* < 0.05; Fig. 2C). Compared with the control group, P2X_4_^−/−^ LDLR^−/−^ mice had lower leukocyte counts before diet, whereas leukocyte counts after diet were slightly higher in the P2X_4_^−/−^ LDLR^−/−^ group (Table 1). In the differential blood count a lower proportion of total t-cells, CD4+ T cells and a higher proportion of CD8+ T cells were phenotypically observed in P2X_4_^−/−^ LDLR^−/−^ mice, both before and after diet (Table 1). Interestingly, the analysis of monocyte subgroups revealed that P2X_4_^−/−^ LDLR^−/−^ mice shared a lower proportion of pro-inflammatory Ly6C^high^ monocytes and a higher proportion of anti-inflammatory Ly6C^low^ monocytes before and after the diet (Table 1). However, no significant differences in the proportion of common myeloid progenitor cells were found in the bone marrow between P2X_4_-deficient and P2X_4_-competent mice (Supplemental Table 1). There were no other differences in the differential blood count, triglycerides and cholesterol. Health status, weight, life span, behavior, and reproduction did not differ between both groups. Altogether, these findings indicate a lower inflammatory burden in P2X_4_^−/−^ animals.

**Table 1:** Baseline characteristics P2X4-Knockout Study.

		LDL-R^−/−^	P2X4^−/−^ LDL-R^−/−^	*p* value
Total leukocytes, tsd/µL	Before diet	5.94 ± 0.35	3.82 ± 0.24	***
	After diet	7.19 ± 0.83	9.5 ± 0.68	*
Neutrophils/leukocytes, %	Before diet	10 ± 1.5	11 ± 1	ns
	After diet	18 ± 1.3	18 ± 1.9	ns
B-cells/leukocytes, %	Before diet	46 ± 1.9	51 ± 2.1	ns
	After diet	43 ± 1.1	42 ± 3.6	ns
T-cells/leukocytes, %	Before diet	23 ± 0.69	17 ± 1.4	**
	After diet	15 ± 1	9.4 ± 0.97	***
CD4+ cells/T-cells, %	Before diet	61 ± 1	48 ± 1.8	****
	After diet	51 ± 2.5	38 ± 3	**
CD8+ cells/T-cells, %	Before diet	39 ± 1	52 ± 1.8	****
	After diet	49 ± 2.2	62 ± 3	**
Monocytes/leukocytes, %	Before diet	4.93 ± 0.57	5.795 ± 0.53	ns
	After diet	11 ± 0.98	13 ± 0.95	ns
Ly6C high monocytes/monocytes, %	Before diet	46 ± 3.1	39 ± 1.1	*
	After diet	65 ± 2.8	54 ± 2.1	**
Ly6C low monocytes/monocytes, %	Before diet	54 ± 3.1	61 ± 1.1	*
	After diet	35 ± 2.8	46 ± 2.1	**
Weight (g)	Before diet	21.06 ± 0.54	21.02 ± 0.28	ns
	After diet	33.79 ± 1.1	35.54 ± 0.94	ns
Total cholesterol (mg/dL)	After diet	2200 ± 190	2271 ± 142	ns
Total triglycerides (mg/dL)	After diet	722 ± 100	607 ± 37	ns

Blood was taken from P2X_4_^−/−^ LDLR^−/−^ mice (n = 24) and P2X_4_^+/+^ LDLR^−/−^ mice (n = 15) before and after 16 weeks of high-cholesterol diet, leukocytes were measured in tsd/ul. Leukocyte subsets were analyzed before and after diet by fluorescence-activated cell sorting. Weight was taken before and after diet in P2X_4_^−/−^ LDLR^−/−^ mice (n = 24) and P2X_4_^+/+^ LDLR^−/−^ mice (n = 15). Plasma total cholesterol and total triglycerides were assessed after diet. Results are presented as mean ± SEM. Statistical significance was calculated using an unpaired t-test for parametric or Mann–Whitney-U-test for non-parametric data.

*p < 0.05; **p < 0.01; ***p < 0.001; ****p < 0.0001.

### P2X_4_ does not influence plaque composition in atherosclerotic lesions

**Figure 3:**
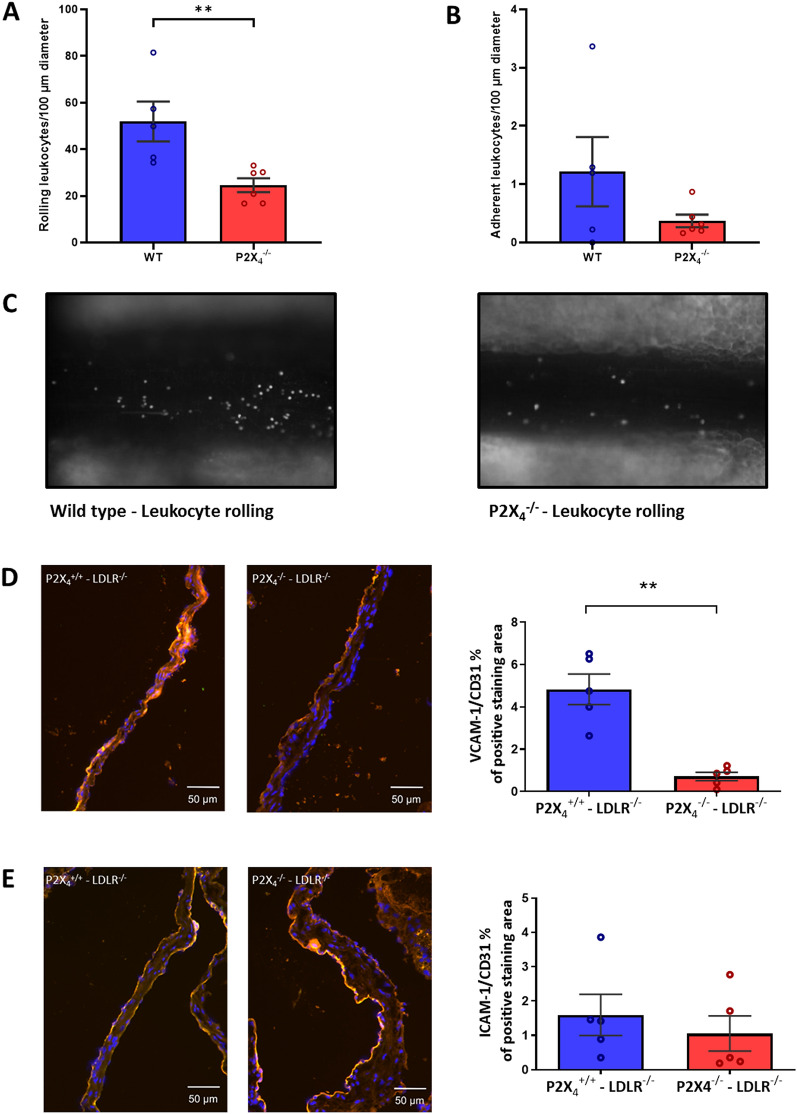
P2X_4_-deficiency limits leukocyte rolling and reduces endothelial VCAM-1 expression. P2X_4_-deficient (n = 6) and P2X_4_–competent mice (n = 5) were intraperitoneally stimulated with ATP. 2 h after stimulation, leukocyte rolling and adhesion was assessed by intravital microscopy (**A**, **B**). Representative images of leukocyte rolling are presented (**C**). Endothelial expression of VCAM-1 (red) (**D**) and ICAM-1 (red) (**E**) was assessed by 3-colour-immunofluorescence with additional stainings for cell nuclei (DAPI, blue) and endothelial cells (anti-CD31, green), representative images of merged sections are shown (**D**, **E**). Statistical significance was calculated using unpaired t-test. **p < 0.01.

Plaque composition plays an important role in advanced human atherosclerotic lesions with regard to possible plaque rupture. Thus, we further assessed crucial features of atherosclerotic plaque composition by histochemistry. Analysis of plaque composition showed no significant differences between P2X_4_^−/−^ LDLR^−/−^ mice and P2X_4_^+/+^ LDLR^−/−^ mice. There was no significant difference in the content of lipids (Oil-red-O-positive area per % of lesion: P2X_4_^+/+^ LDLR^−/−^: 25.53 ± 11.73%, n = 14; P2X_4_^−/−^ LDLR^−/−^: 22.35 ± 9.93%, n = 24; n.s.; Supplemental Fig. 1A and 1B) and macrophages (MAC-3-positive area per lesion: P2X_4_^+/+^ LDLR^−/−^: 22.31 ± 6.92%, n = 14; P2X_4_^−/−^ LDLR^−/−^: 23.76 ± 8.37%, n = 24; n.s.; Supplemental Fig. 1A and 1C) within the lesions of both groups. Moreover plaques of both groups showed similar contents of smooth muscle cells (α-actin -positive area per lesion: P2X_4_^+/+^ LDLR^−/−^: 6.57 ± 3.45%, n = 14; P2X_4_^−/−^ LDLR^−/−^: 6.05 ± 4.23%, n = 24; n.s.; Fig. 3A,D) and collagen (Sirius red-positive area per lesion: P2X_4_^+/+^ LDLR^−/−^: 6.32 ± 4.55%, n = 14; P2X_4_^−/−^ LDLR^−/−^: 4.79 ± 4.31%, n = 21; n.s.; Supplemental Fig. 1A and 1E). Also, the lesional compound of CD4^+^ cells was not significantly altered (CD4-positive area per lesion: P2X_4_^+/+^ LDLR^−/−^: 3.09 ± 2.25%, n = 14; P2X_4_^−/−^ LDLR^−/−^: 4.05 ± 2.2%, n = 23; n.s.; Supplemental Figs. 2A and 2B). In addition, the lesions of both groups showed similar proportions of necrotic core areas (Necrotic core area per lesion: P2X_4_^+/+^ LDLR^−/−^: 8.05 ± 1.63%, n = 7; P2X_4_^−/−^ LDLR^−/−^: 7.51 ± 4.2, n = 24; n.s.; Supplemental Figs. 2C and 2D).

### P2X_4_-deficiency limits leucocyte rolling on the vessel wall

Leukocyte recruitment represents an important step in atherogenesis. Therefore, we investigated the influence of P2X_4_ on leukocyte rolling and leukocyte adhesion in vivo. P2X_4_-deficient (n = 6) and P2X_4_-competent mice (n = 5) were stimulated via intraperitoneal ATP injections. Afterwards, leukocyte rolling and adhesion in mesenterial venules was analyzed by intravital microscopy. Leukocyte rolling was significantly impaired in P2X_4_-deficient mice (*p* < 0.01, Fig. 3A,C). However, no significant differences were found in leukocyte adhesion (Fig. 3B,C).

### P2X_4_-deficiency reduces expression of endothelial adhesion molecules

**Figure 4:**
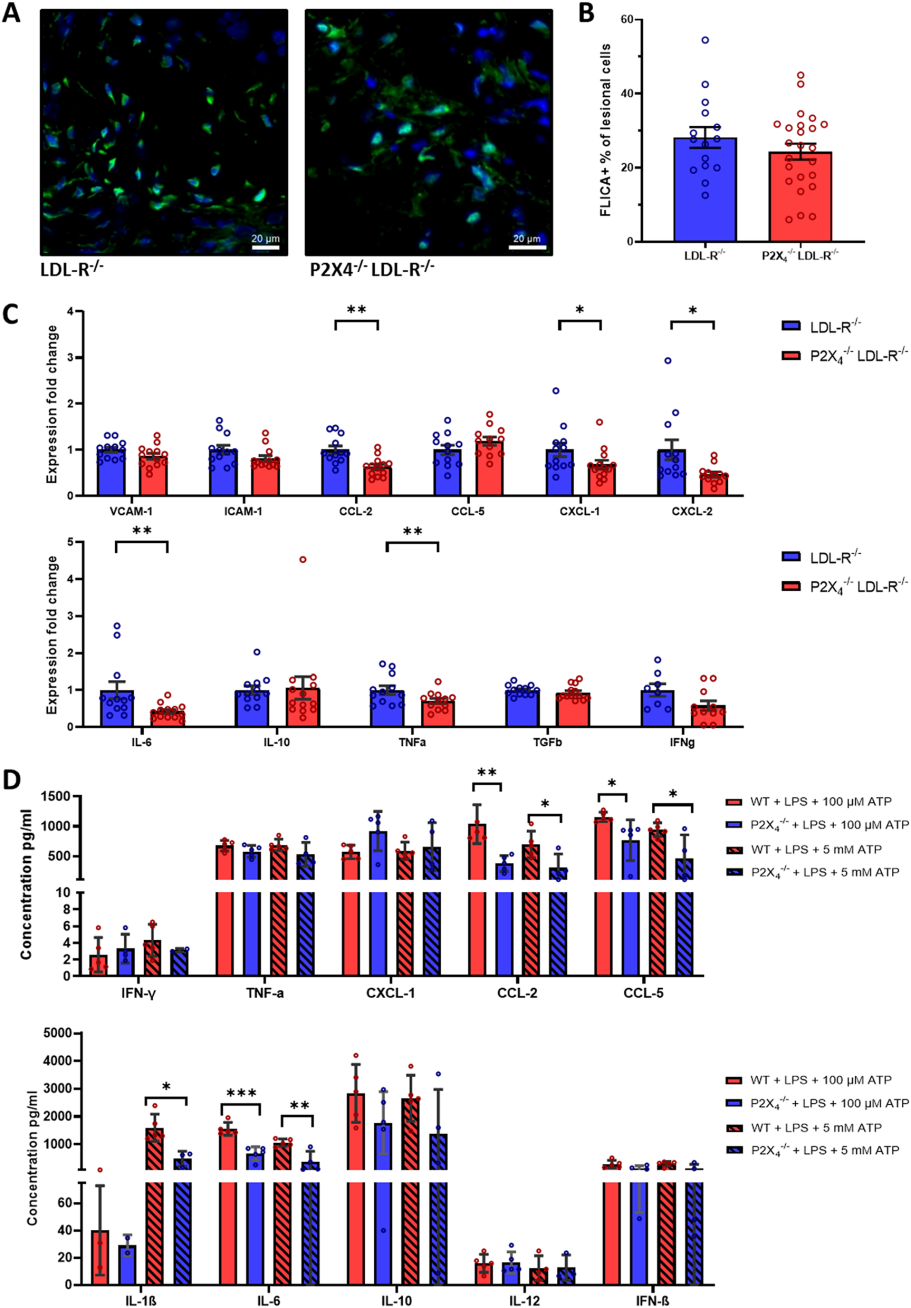
P2X_4_-deficiency reduces expression release of inflammatory cytokines. Atherosclerotic aortic roots from P2X_4_^−/−^ LDLR^−/−^ mice (n = 24) and P2X_4_^+/+^ LDLR^−/−^ mice (n = 15) after 16 weeks of high-cholesterol diet were stained for FLICA-positive cells (FLICA-fmk, green) and cell nuclei (DAPI, blue). Representative images are shown (**A**). Frequency of FLICA-positive cells per DAPI-positive cells was analyzed (**B**). RNA was isolated from atherosclerotic lesions from aortic arches of P2X_4_^−/−^ LDLR^−/−^ mice (n = 12) and P2X_4_^+/+^ LDLR^−/−^ mice (n = 13). Two-step multiplex TaqMan RT-PCR was performed to determine expression of cytokines and adhesion molecules. Expression fold change was calculated by ddCt method, results were referred to β-Actin as the housekeeping gene (**C**). BMDMs were isolated from the bone marrow of 8-week-old P2X4-competent (n = 5) and P2X4-deficient mice (n = 5). Fully differentiated BMDMs were first stimulated with 100 ng/mL LPS for 4 h, followed by stimulation with either 100 µM or 5 mM ATP for 1 h. Multiplex fluorescence-encoded beads assay was performed to determine concentrations of inflammatory cytokines (**D**). Results are presented as mean ± SEM. Statistical significance was calculated using Shapiro–Wilk Test followed by an unpaired t-test for parametric or Mann–Whitney-U-test for non-parametric data. *p < 0.05; **p < 0.01; ***p < 0.001.

For further examination of the endothelial vascular cell adhesion molecule 1 (VCAM-1) and intercellular cell adhesion molecule 1 (ICAM-1) expression, 3-colour-immunofluorescence with additional stainings for cell nuclei (DAPI) and endothelial cells (anti-CD31) was performed. Immunohistochemistry demonstrated a reduced expression of endothelial VCAM-1 in P2X_4_^−/−^ LDLR^−/−^ mice (n = 5), compared with P2X_4_^+/+^ LDLR^−/−^ mice (n = 5; *p* < 0.01, Fig. 3D). The endothelial expression of ICAM-1 was similar between both groups (Fig. 3E). Opposed to the endothelial expression, the lesional expression of VCAM-1 and ICAM-1 did not differ between P2X_4_^−/−^ LDLR^−/−^ (n = 12) and P2X_4_^+/+^ LDLR^−/−^ (n = 13) mice according to real-time polymerase chain reaction (Fig. 4C).

### P2X_4_-deficiency reduces inflammasome priming, expression, and release of inflammatory cytokines

P2X_4_ is known to mediate the release of cytokines such as IL-6 and TNFα in vivo and was shown to promote proximal tubular NLRP3-inflammasome activation in vivo^24,28^. Because of the important role of the NLRP3-inflammasome in the onset and maintenance of cardiovascular inflammation, we assessed the extend to which inflammasome-activation is influenced in atherosclerotic plaques in P2X_4_-deficient mice. Immunohistochemically, no differences in the proportion of FLICA-positive cells – a staining for active caspase 1—in P2X_4_-deficient and P2X_4_-competent mice could be detected (FLICA-positive area per lesion: P2X_4_^+/+^ LDLR^−/−^: 28.19 ± 10.94%, n = 15; P2X_4_^−/−^ LDLR^−/−^: 24.37 ± 10.53%, n = 24; n.s.; Fig. 4A,B). To investigate molecular expression levels of inflammatory cytokines and inflammasome priming, RNA was isolated from atherosclerotic lesions from aortic arches after 16 weeks of high-cholesterol diet and real-time polymerase chain reaction was performed. Interestingly, atherosclerotic lesion from P2X_4_^−/−^ LDLR^−/−^ mice (n = 12) showed significantly lower priming of NLRP3, compared with P2X_4_^+/+^ LDLR^−/−^ mice (n = 13) (*p* < 0.05, Fig. 4C). Regarding cytokines, expression levels of CC chemokine ligand 2/monocyte chemotactic protein 1 (CCL-2/MCP-1, *p* < 0.01), C-X-C motif chemokine-1 (CXCL-1, *p* < 0.05), C-X-C motif chemokine-2 (CXCL-2, *p* < 0.05), IL-6 (*p* < 0.01) and tumor necrosis factor α (TNFα, *p* < 0.01) were significantly decreased in atherosclerotic plaques from P2X_4_^−/−^ LDLR^−/−^ mice (Fig. 4C). In contrast, no differences were found in the expression levels of IL-1β, CC chemokine ligand 5 (CCL-5), Interleukin-10 (IL-10) and transforming growth factor β (TGF β) (Fig. 4C).

To further clarify the influence of P2X_4_^−/−^ on plasma cytokine concentrations, plasma from euthanized P2X_4_^−/−^/LDLR^−/−^ and LDLR^−/−^ male mice after 16 weeks of high-cholesterol diet was analyzed by a multiplex assay utilizing fluorescence-encoded beads. P2X_4_^−/−^ LDLR^−/−^ mice (n = 5) showed a lower concentration of IL-12 (*p* < 0.05) in plasma compared with P2X_4_^+/+^ LDLR^−/−^ mice (Supplemental Fig. 4A). Beyond that, there were no differences in plasma concentrations of TNF-α, IFN-β, IFN- γ, IL-1β, IL-6, IL-10, CCL-2, CCl-5, and CXCL-1 between P2X_4_^−/−^ LDLR^−/−^ mice and P2X_4_^+/+^ LDLR^−/−^ mice (Supplemental Fig. 4A).

For further mechanistic clarification of the abovementioned cytokine expression data from atherosclerotic aortic arch lesions after 16 weeks of high-cholesterol diet, BMDMs were isolated from P2X_4_-competent (n = 5) and P2X_4_-deficient mice (n = 5) (Fig. 4D). Subsequently, fully differentiated BMDMs were first stimulated with 100 ng/mL LPS for 4 h, followed by stimulation with either 100 µM ATP or 5 mM ATP. Cytokine levels were determined in the cell culture supernatant one hour after ATP stimulation. Both after stimulation with 100 µM ATP and after stimulation with 5 mM ATP, significantly reduced concentrations of CCL-2 (1 h after 100 µM ATP: *p* < 0. 01; 1 h after 5 mM ATP: *p* < 0.05), CCL-5 (1 h after 100 µM ATP: *p* < 0.05; 1 h after 5 mM ATP: *p* < 0.05) and IL-6 (1 h after 100 µM ATP: *p* < 0.001; 1 h after 5 mM ATP: *p* < 0.01) were detected in the supernatant of P2X_4_-deficient BMDMs (Fig. 4D). In addition, after stimulation with 5 mM ATP, significantly reduced concentrations of IL-1β were measured in the supernatant of P2X_4_-deficient BMDMs (1 h after 5 mM ATP: *p* < 0.05). After stimulation with 100 µM ATP, no difference was found in the concentrations of IL-1β between P2X_4_-competent and P2X_4_-deficient mice (Fig. 4D).

### P2X_4_ is expressed in human atherosclerotic lesions

Since our data indicate an important role of P2X_4_ in experimental atherosclerosis, we further evaluated whether P2X_4_ is expressed in human atherosclerotic plaques. Human atherosclerotic lesions from carotid endarterectomy (n = 10) were stained against P2X_4_ for immunohistochemical analysis of P2X_4_ expression. P2X_4_ expression was significantly increased in atherosclerotic-diseased vessel areas (n = 10), compared to non-atherosclerotic parts of the vessel wall (n = 7, *p* < 0.05, Figure Supplemental 5A and 5B). As shown by the 3-colour immunofluorescence staining, P2X_4_ was particularly colocalized with CD31 positive endothelial cells in human atherosclerotic plaques (Supplemental Fig. 5C). These data suggest that P2X_4_ is involved in human atherosclerotic disease.

## Discussion

This study demonstrated for the first time that P2X_4_-deficiency reduces atherosclerosis and diminishes vascular inflammation. Various studies identified extracellular ATP to mediate inflammation by inducing the release of various pro-inflammatory chemokines through activation of P2X_4_^23,29,30^. Since P2X_4_ expression on cell surfaces is particularly sensitive being upregulated by stress stimuli, ischemia or inflammation, P2X_4_ expression has been described in numerous chronic inflammatory diseases on stromal cell types including endothelial and epithelial cells^18,19^. Indeed, we found an increased expression of P2X_4_ in atherosclerotic lesions histologically as well as on the RNA level. In line with the literature, 3-colour immunofluorescence indicated that P2X_4_ is particularly colocalized with endothelial cells, which in turn have an important function in atherogenesis.

Further, P2X_4_^−/−^ LDLR^−/−^ mice developed significantly smaller atherosclerotic lesions, indicating a functional role of P2X_4_ in atherogenesis. Beyond decreased lesion size, atherosclerotic lesions from P2X_4_^−/−^ LDLR^−/−^ mice revealed substantially less plaque inflammation. In fact, mechanistically, we found a significantly lower expression of the pro-inflammatory cytokines CCL-2/MCP-1, CXCL-1, CXCL-2, IL-6 and TNFα at mRNA level in atherosclerotic lesions of P2X_4_^−/−^ LDLR^−/−^ mice. It is well established that these cytokines exhibit highly pro-atherogenic properties^31,32^. Supporting our findings, P2X_4_-depression was shown to decrease serum levels of TNF-α and IL-6 in a mouse model of collagen-induced arthritis^24^. Furthermore, it could be shown that ATP-mediated activation of P2X_4_ induced the release of C-X-C motif chemokine-5 (CXCL-5) in primary monocyte‐derived human macrophages^33^. Besides the expression of the pro-inflammatory cytokines in plaque, we observed reduced IL-12 levels in the plasma of P2X_4_^−/−^ LDLR^−/−^ mice in our in vivo study. Consistent with our results, previous studies demonstrated that functional blockade of endogenous IL-12 resulted in a significant decrease in atherogenesis in LDLR^−/−^ mice^34^. In addition to our in vivo data, we were able to demonstrate a reduced ATP-mediated release of the pro-inflammatory cytokines CCL-2, CCL-5, IL-6 and IL-1ß by BMDMs from P2X_4_-deficient mice in vitro. Although P2X_4_-deficient bone marrow-derived dendritic cells revealed decreased IL-1ß secretion as demonstrated an in vitro model of chronic respiratory inflammation and our in vitro model indicated a reduced release of IL-1ß in BMDMs, in our in vivo study, we could not observe a reduction of IL-1ß expression in atherosclerotic lesions from P2X_4_^−/−^ LDLR^−/−^ mice^35^. Considering these data, the pro-atherogenic effects of P2X_4_ are most likely to be explained by the modulation of lesional inflammatory cytokine expression. Although no differential plaque proportions of inflammatory cells were detected in vivo, the cytokine phenotype observed among P2X4-deficient mice suggests that at least parts of the anti-inflammatory effects may be related to reduced macrophage-mediated cytokine and chemokine release. Finally, the P2X_4_-mediated, limited inflammatory activity in the plaque microenvironment may explain an overall decreased atherogenesis with unaltered plaque composition.

NOD-like receptor 3 (NLRP3) inflammasome mediated inflammation is crucially involved in modulating atherosclerotic plaque progression^36^. ATP-P2X_4_ signaling was described to stimulate the activation of the NLRP3 inflammasome in podocytes, increasing the expression and release of IL-1β and Interleukin-18 thereby causing tubular necrosis and aggravating renal fibrosis^22,28^. In our study, NLRP3 inflammasome priming was reduced on the RNA level in atherosclerotic lesions of P2X_4_^−/−^ LDLR^−/−^ mice. Overall, in atherosclerosis P2X_4_ may be involved in the initiation of inflammasome priming, but does not appear to exert a pronounced influence on inflammasome assembly since we did not find difference in IL-1β RNA expression and caspase 1 activity between P2X_4_-deficient and P2X_4_-competent mice as assessed by FLICA staining. Compared to the P2X_7_ receptor, an ATP-responsive ligand gated ion channel playing an important role in atherogenesis via mediating NLRP3 inflammasome activation, P2X_4_ has similar characteristics, but a lower Ca^2+^ flow due to a smaller ion channel size^33,37^. Interestingly, the literature describes the formation of a heterotrimer from P2X_4_ and P2X_7_^38^. Corresponding to this, in our in vitro study, no difference in the release of IL-1β was observed after stimulation of BMDMs from P2X_4_-deficient and P2X_4_-competent mice with a dose of 100 µM ATP. After stimulation with a higher dose of 5 mM ATP, we found a significantly lower release of IL-1β by BMDMs from P2X_4_-deficient mice. In this context, a stimulation dose of 100 µM ATP dose is merely able to activate the more sensitive P2X_4_ receptor alone, whereas with the stimulation dose of 5 mM ATP the receptor P2X_7_ is also activated. In the light of these data, it can be assumed that the P2X_4_ receptor alone may not be able to influence the secretion of IL-1β. Due to the significantly higher sensitivity of P2X_4_ concerning their common ligand ATP, a possible initiation of the inflammatory response including inflammasome priming through P2X_4_ and subsequent pronounced mediation of inflammasome activation with the secretion of IL-1β by P2X_7_ is conceivable^39–41^.

While Ly6C^high^ monocytes promote inflammation through the secretion of pro-inflammatory cytokines, Ly6C^low^ monocytes are associated with anti-inflammatory properties^42,43^. Both at baseline and after a high cholesterol diet, P2X_4_-deficient mice were found to share a significantly lower proportion of pro-inflammatory Ly6C^high^ monocytes and at the same time a higher amount of anti-inflammatory Ly6C^low^ monocytes. At the same time, in the bone marrow, no differences in common myeloid progenitor cells were detected between P2X_4_-deficient and P2X_4_-competent mice. Overall, these results are most likely to be associated with systemic anti-inflammatory properties of P2X_4_-deficiency with lower expression of pro-inflammatory cytokines in atherosclerotic plaques and limited secretion of pro-inflammatory cytokines by BMDMs from P2X_4_-deficient mice^44^.

Because leukocyte recruitment plays a central role in atherogenesis, the influence of P2X_4_ was characterized by intravital microscopy. In this study, P2X_4_-deficient mice were found to show a tendency towards limited leukocyte rolling. This is in line with the reduced lesional expression of the chemoattractants CCL-2, CXCL-1, CXCL-2 as well as the decreased concentrations of the chemotractants CCL-2 and CCL-5 in BMDM supernatant and an overall lower inflammatory activation and thereby potentially lower leukocyte reactivity^45^. In contrast, no effect of P2X_4_-deficiency on leukocyte adhesion to the vessel wall was observed. Mechanistically, we found a reduced expression of the endothelial adhesion factor VCAM-1 in P2X_4_-deficient mice. VCAM-1 promotes the adherence of leukocytes to endothelial cells, whereas stimulation with ATP is known to increase the expression of endothelial VCAM-1^46,47^. Although there were no differences in lesional expression of adhesion molecules within plaques between P2X_4_ deficient mice and controls, the main effect of VCAM-1 on vascular adhesion of leukocytes is mostly related to endothelial cells^47^. Therefore, the decreased endothelial expression of VCAM-1 may explain, at least in part, the tendency toward limited leukocyte rolling. Nethertheless, because of the statistically significant but rather mild effect of leukocyte rolling and the unaffected plaque composition, reduced leukocyte rolling may only contribute to a part of the observed anti-atherogenic effects in P2X4 deficiency.

Additionally, P2X_4_-deficient mice, both before and after diet, presented a lower proportion of total T cells, CD4+ cells and a higher proportion of CD8+ cells. Despite the differences in blood count, immunohistochemistry showed no P2X_4_-mediated effects on T-cells in atherosclerotic plaques. Consequently, a P2X_4_-specific blood phenotype is most likely to be assumed in this context.

Previous studies described the endothelial expression of P2X_4_ and P2X_4_-mediated pro-inflammatory responses in human umbilical vein endothelial cells in vitro^23^. In line with this, we were able to demonstrate the involvement of P2X_4_ in human atherosclerotic plaques for the first time. Consistent with the literature and our histochemical data from the murine atherosclerosis study, P2X_4_ is also found in human atherosclerotic plaques to be predominantly colocalized with endothelial cells^23^. Since P2X_4_ is also present in human atherosclerotic plaques, the results of this study could potentially be applicable to human atherosclerotic diseases and could make an important contribution to further research of new treatment options.

In conclusion, P2X_4_-deficiency was shown to improve atherosclerosis, reduced the expression and the release of pro-inflammatory cytokines and decreased inflammasome priming. Consequently, P2X_4_ appears to be a promising target structure for the treatment of atherosclerosis and cardiovascular inflammation.

## Supplementary Information

Supplementary Information 1.

Supplementary Information 2.

## Data Availability

Primary data will be made available upon request.
