# Supplementary material for: P2X4 deficiency reduces atherosclerosis and plaque inflammation in mice
Source: Sci Rep. 2022 Feb 18;12:2801. doi: 10.1038/s41598-022-06706-6 (PMC8857235; doi:10.1038/s41598-022-06706-6)
Supplement: Supplementary file 1 — Supplementary Information 1. [file 41598_2022_6706_MOESM1_ESM.docx]

**Supplemental Figures**

**Supplemental Figure 1.**

**
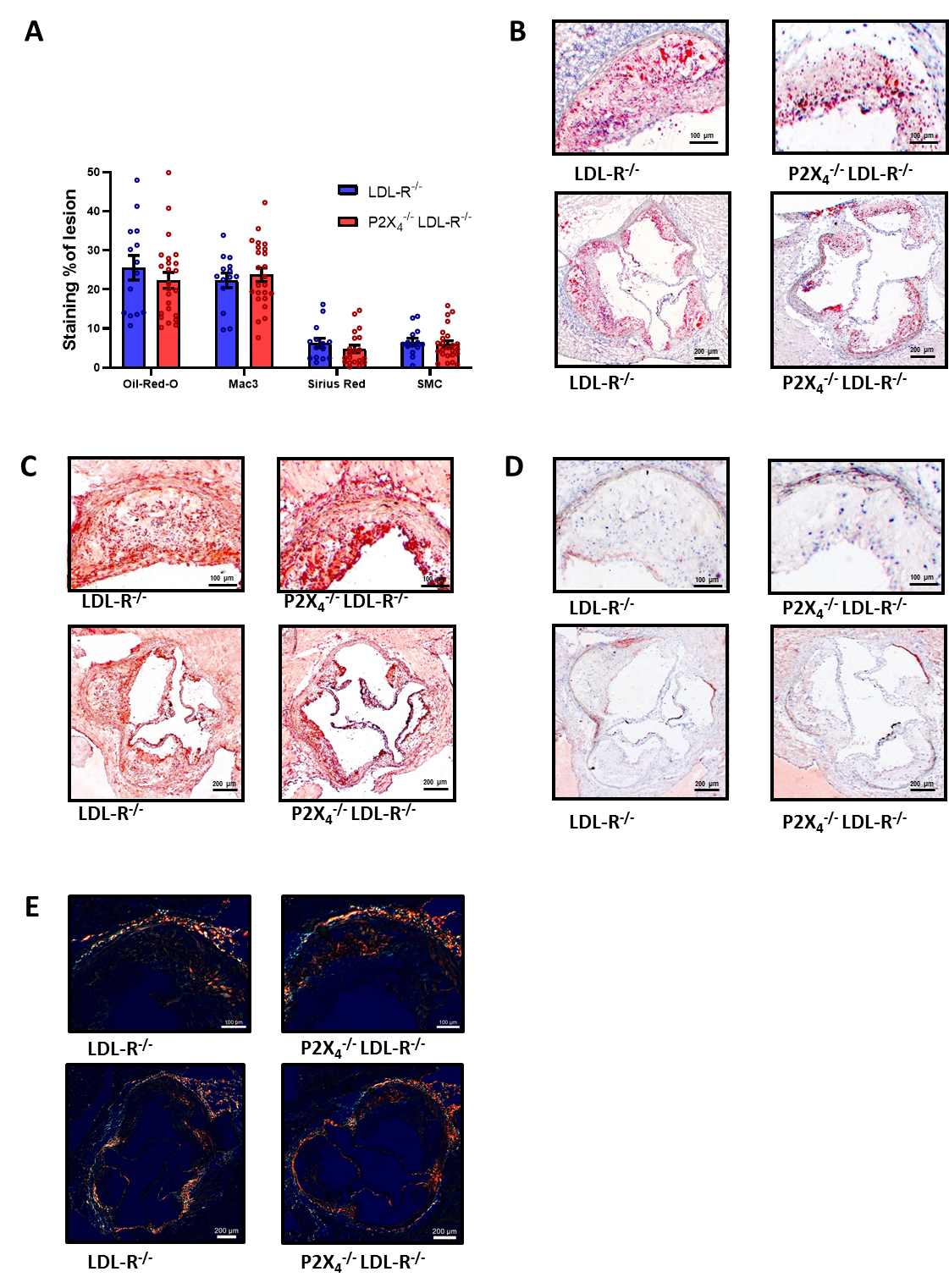
**

**Supplemental Figure 2.**

**
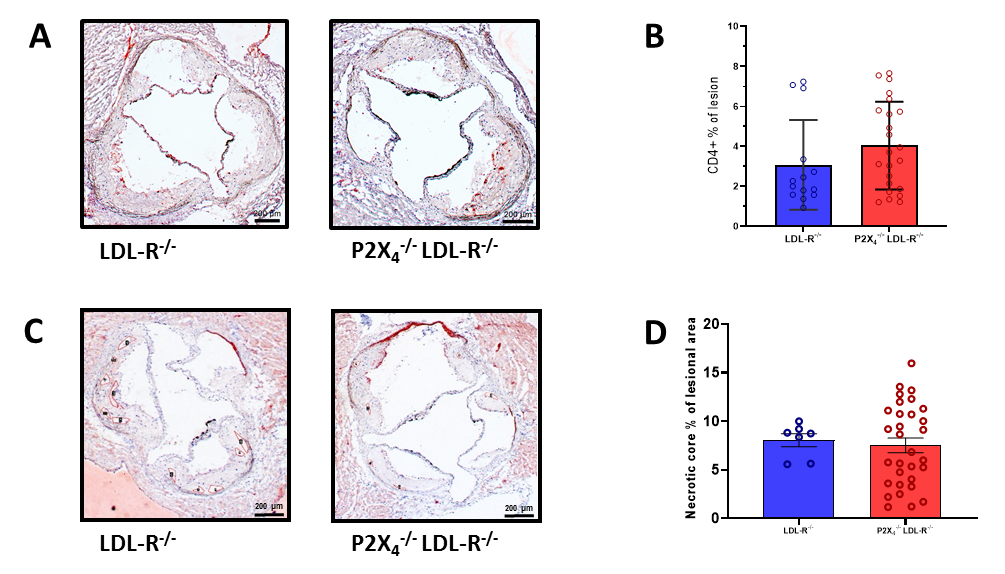
**

**Supplemental Figure 3.**

**
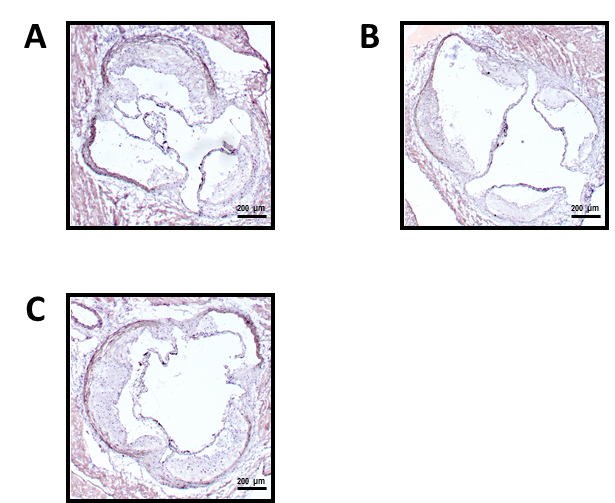
**

**Supplemental Figure 4.**


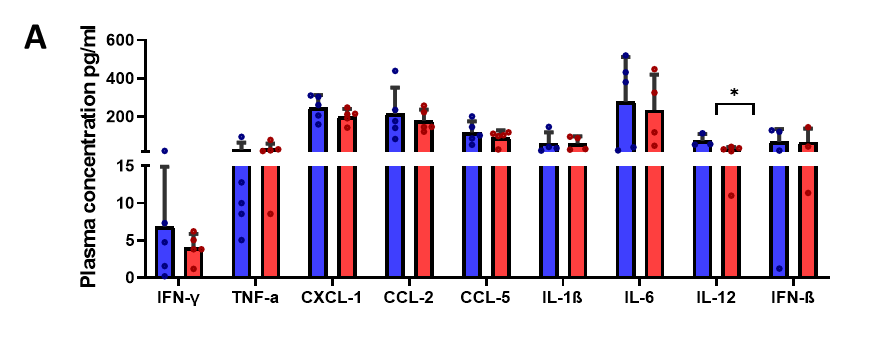


**Supplemental Figure 5.**


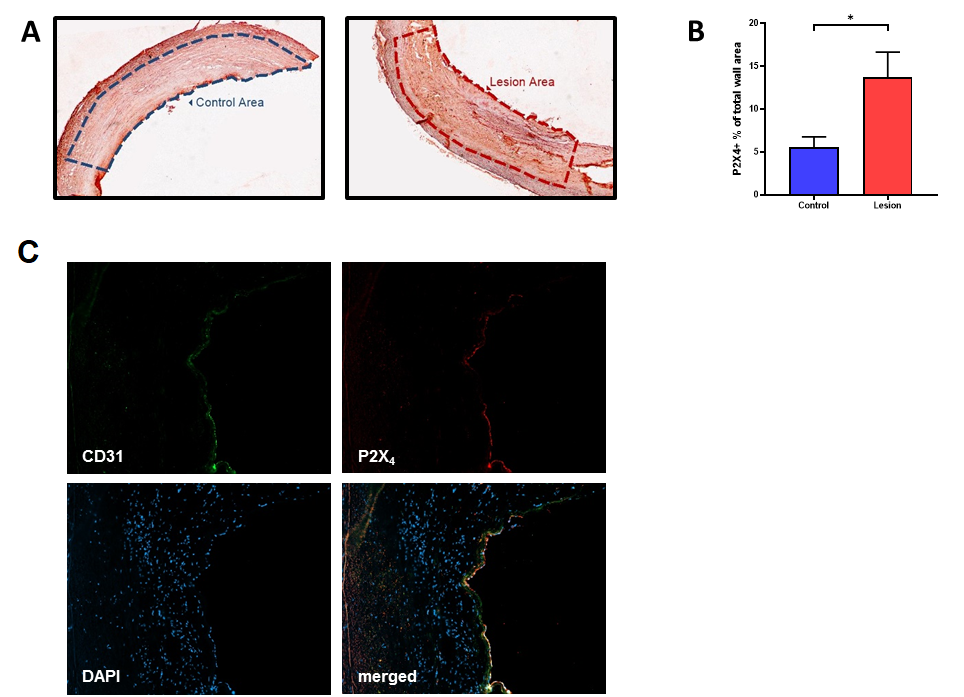


**Figure legends**

**Supplemental Figure 1. P2X_4_ does not influence plaque composition in atherosclerotic lesions.** After 16 weeks of high-cholesterol diet, aortic arches from P2X_4_^-/-^ LDLR^-/-^ mice (n=24) and P2X_4_^+/+^ LDLR^-/-^ mice (n=14) were stained for lipids (Oil-red-O, **A** and **B**), macrophages (P2X_4_^-/-^ LDLR^-/-^ (n=24) and P2X_4_^+/+^ LDLR^-/-^ (n=14); anti-Mac-3, **A** and **C**), smooth muscle cells (P2X_4_^-/-^ LDLR^-/-^ (n=24) and P2X_4_^+/+^ LDLR^-/-^ (n=14);anti –α-actin, **A** and **D**) and collagen (P2X_4_^-/-^ LDLR^-/-^ (n=21) and P2X_4_^+/+^ LDLR^-/-^ (n=14);Sirius red, **A** and **E**), representative images are shown. Results are presented as mean±SEM. Statistical significance was calculated using unpaired t-test (parametric) or Mann-Whitney*-U*-test (non-parametric data).

**Supplemental Figure 2. P2X_4_ does not influence plaque content of CD4+ cells and necrotic core area size in atherosclerotic lesions.** After 16 weeks of high-cholesterol diet, aortic arches from P2X_4_^-/-^ LDLR^-/-^ mice (n=23) and P2X_4_^+/+^ LDLR^-/-^ mice (n=14) were stained for CD4^+^ cells (anti-CD4, **A** and **B**), representative images are shown. Necrotic core size was analyzed by measuring acellular intima lesion areas in anti-α- actin stained sections of the aortic root (**C** and **D**). Results are presented as mean±SEM. Statistical significance was calculated using unpaired t-test (parametric) or Mann-Whitney test (non-parametric data).

**Supplemental Figure 3. Immunohistochemistry negative controls.** After 16 weeks of high-cholesterol diet, aortic arches from P2X_4_^-/-^ LDLR^-/-^ mice (n=23) and P2X_4_^+/+^ LDLR^-/-^ mice (n=14) were stained by immunohistochemistry for macrophages (anti-Mac-3), smooth muscle cells (anti –α-actin), collagen (Sirius red) and CD4^+^ cells (anti-CD4). Representative images from negative controls of stainings with anti-Mac-3 (**A)**, anti –α-actin (**B**), and anti-CD4 (**C**) are shown.

**Supplemental Figure 4. Determination of plasma cytokine concentrations.**

Cytokine concentrations were analyzed in plasma from P2X4^-/-^ / LDLR^-/-^ (n=5) and LDLR-/- (n=5) mice after 16 weeks of high-cholesterol diet by a multiplex assay utilizing fluorescence-encoded beads. Cytometric analysis cytokine concentrations were performed (**A**). Results are presented as mean ±SEM. Statistical significance was calculated using unpaired t-test for parametric or Mann-Whitney-U-test for non-parametric data. *p<0.05

**Supplemental Figure 5. P2X_4_ expression is increased in human atherosclerotic plaques.** Human plaques from carotid thrombendarteriectomy (n=10) were stained with anti-P2X4 for immunohistochemistry. Representative images of human carotid lesions are shown (A). P2X4-positive areas were quantified and compared in atherosclerotic lesion areas (n=10) and unaffected control areas (n=7) of the same vessel (A and B). The distribution of P2X4 in human carotid lesions was analyzed by 3-colour immunofluorescence staining for cell nuclei (DAPI, blue), endothelial cells (CD 31, green), and P2X4 (red). Representative sections are shown in the merged images (C). Results are presented as mean±SEM. Statistical significance was calculated using nonparametric Mann-Whitney*-U*-test. *p<0.05.

**Supplemental Tables**

**Supplemental Table 1. Common myeloid progenitor cells analysis.**

|  | WT | P2X_4_^-/-^ | P Value |
| --- | --- | --- | --- |
| Common myeloid progenitors [% of BM leukocytes] | 2,1 ± 0,27 | 2,67 ± 0,44 | ns |

Hematopoietic progenitor cells were isolated from the bone marrow of 8-week-old P2X_4_-competent (n=3) and P2X_4_-deficient mice (n=3) as previously described. Common myeloid progenitor cells were analyzed by fluorescence-activated cell sorting based on CD45^+^, Lin^-^ (CD3^-^, CD19^-^, NK1.1^-^, Gr-1^-^, CD 11b^-^, and Il7Ra^-^), cKit^+^, and Sca-1^-^ cell surface phenotypes. Results are presented as mean ± SEM.
