# Supplementary material for: P2X4 deficiency reduces atherosclerosis and plaque inflammation in mice
Source: Sci Rep. 2022 Feb 18;12:2801. doi: 10.1038/s41598-022-06706-6 (PMC8857235; doi:10.1038/s41598-022-06706-6)
Supplement: Supplementary file 2 — Supplementary Information 2. [file 41598_2022_6706_MOESM2_ESM.docx]

**Histochemistry**

**Oil Red O staining**

For histochemical staining of lipids 0.5% Oil-red-O solution was prepared one day before the staining procedure. Therefore 2.5 g pure Oil red O (Sigma-Aldrich, St. Luis, MO, USA) were solved under stirring in 500ml propylene glycol (1,2- Propandiol, Fisher Scientific, Waltham, MA, USA) at 95°C. The obtained suspension was filtered through 185mm filter paper (Whatman GmbH, Dassel, Germany) and cooled down to room temperature. Before staining, filtration was repeated with a 0.2µm filter (Nalgene vacuum filtration system, SigmaAldrich, St. Luis, MO, USA). Sections of the aortic root and arch were adapted to room temperature. Afterwards they were fixed for 10min in 10% formalin. After washing under floating water dehydration was performed in 100% propylene glycol. Sections were stained in 0.5% Oil Red O for 25min at 60°C. After 10min washing under floating water cell nuclei were counterstained for 5sec in 25% hematoxylin (Sigma-Aldrich, St. Luis, MO, USA) and dipped 0.25% ammonium (Ammonia water 0.25%, Electron Microscopy Sciences, Hatfield, PA, USA). Finally they were embedded in glycerol gelatin (Sigma-Aldrich, St. Luis, MO, USA) and covered with a cover slip.

**Picrosirius Red staining**

For histological staining of collagen, a 0.1% solution of sirius red powder (Polyscience inc., Warrington, PA, USA) was prepared in saturated aqueous picric acid (Ricca Chemical Company, Arlington, TX, USA) and filtered. Slides were air dried and fixed in 10% buffered formalin for 10 min at RT. After rinsing with tap water, the slides were incubated for 3-4 hours in picrosirius red solution. Later, rinsed twice for 1 min in 0.01 N HCl, the slides were dehydrated in different concentrations of Ethanol (70% ethanol for 30-45 sec, 95% ethanol about 5min, and 100% ethanol about 5 min) and xylenes (5 min).

**Anti-Mac-3 and α-actin staining**

For immunohistochemical staining of Mac-3 (Anti- Mac-3 (rat anti-mouse), BD Pharmingen, Franklin Lakes, NJ, USA) and α-actin (Anti-actin, α-Smooth Muscle-FITC conjugate , Sigma-Aldrich, St. Luis, MO, USA), sections of the aortic arch and root were adapted to RT and fixed in acetone for 9min. To avoid leak of staining roots and arches were surrounded with polysiloxane (15% Dimethylpolysiloxane, Sigma-Aldrich, St. Luis, MO, USA), 84% propanol(Iso-propyl-alcohol, VWR International GmbH, Darmstadt, Germany) 1% H2SO4(Fisher Scientific, Schwerte, Germany at 65°C. Sections were incubated in 0.3% H2O2 (EMD Chemicals, Merck KGaA, Darmstadt, Germany, # HX0635-1) for 15min at RT. After 3 times washing with PBS for 5min at RT, anti-Mac-3 sections were incubated with 50µl 5% rabbit serum (Vector Laboratories, Burlingame, CA, USA) for 20 min, while a-actin sections were incubated with 50 µl horse serum(Vector Laboratories, Burlingame, CA, USA) for 20min, to avoid unspecific binding. According to the manufacturer’s protocol, serum was removed and staining was performed with the first antibody. After three times washing with PBS for 5min at RT, anti-Mac-3 sections were overlaid with biotinylated Rabbit anti-Rat (Vector Laboratories, Burlingame, CA, USA) for 45 min at RT, while a-actin sections were overlaid with anti-FITC-biotin conjugated (Sigma-Aldrich, St. Luis, MO, USA) for 45 min at RT. This step was followed by 3 times washing with PBS for 5min at RT. Sections were subsequently incubated with 50µl of Elite PK-6100 Vectastain ABC kits (Vector Laboratories, Burlingame, CA, USA) for 30min at RT. After washing with PBS 3 times for 5min at RT, staining was developed with a drop of Chromagen AEC Substrat (DAKO, Hamburg, Germany) for 1 to 3min. Stained sections were washed for 20min under floating water and cell nuclei were counterstained with hematoxylin.

**VCAM-1 and ICAM-1 staining**

4-µm thick serial cryostat sections of the aortic root were obtained following perfusion of the mice using PBS-/-. Sections were initially blocked using CAS-Block (008120; life technologies, Frederick, MD, USA) and then incubated with anti-CD31 (1:200) (ab28364; abcam, Cambridge, UK) and CD102 (ICAM-1;1:200) (553325; BD Pharmingen, San Diego, CA, USA) or CD106 (VCAM-1;1:200) (553330; BD Pharmingen, San Diego, CA, USA) overnight at 4°C. The secondary antibodies used for our double immunofluorescence were alpaca anti-rabbit IgG Alexa 488 (1:1000) (srbAF488-1; chromotek, Planegg, Bavaria) and rabbit anti-rat IgG Secondary Antibody, TRITC (1:200) (PA1-28570; Invitrogen, OR, USA). All slides were mounted with VectaShield mounting medium with DAPI (H-1200-10; Vector Laboratories, Burlingame, California, USA) and micrographs taken (Axio Imager. Z2; Zeiss).

**Quantitative analysis of sections of the aortic root**

Image pro computer software (Image pro, Cybernetics, Bethesda, USA) is used to evaluate the aortic preparations. For quantitative measurement of the aortic sections, a standard is determined for the selected 4x magnification. Initially, the "total area" of the vessel diameter is determined by tracing the interface between adventia and media. Thereupon, the luminal interface of the intima is traced around to obtain the "intima" area. If subtracted from the "total area", the area of the vessel wall, the "total wall area" (TWA), is obtained. By tracing the interface between media and intima, the software calculates the area "Media". Subtracting the "Media" area from the "Total Area" results in the media area of the aortic wall. Furthermore, subtracting the media area from the total wall diameter (TWA) results in the area of the vascular intima including potential plaques. In this context, the size of the intimal area is proportional to the size of atherosclerotic plaques. From the measurement results of the respective areas as well as staining, mean values are subsequently calculated individually for each animal.

The evaluation of the staining for smooth muscle cells, collagen, lipids and macrophages is performed according to the same method. A color recognition tool is used to determine a specific hue as positive for the associated staining. All points of the selected color are evaluated by the program as positive, so that the software calculates the positive area. To limit the measurement area, an "Area Of Interest" (AOI) is determined in order to exclusively measure the positive hues within this defined area. The "Area Of Interest" was defined as the "Total Wall Area" and the area of the intima. The measurements are then considered in relation to the averaged TWA and intima areas, respectively.

**Quantitative analysis of sections of the aortic arches and abdominal aortas**

To evaluate the sections of the aortic arches and abdominal aortas, it is necessary to clearly determine the area to be measured in order to generate comparable results. For the aortic arches, the area defined as "Area Of Interest" starts behind the 3rd branch of the aortic arch (Arteria subclavia sinistra). From this point, it initially continues in the direction of the convex side of the aortic arch, from where again 2mm is measured in the direction of the 1st outlet of the aortic arch. Within this area, TWA, media, and intima are measured, and the corresponding staining within the "AOI" is quantified. For abdominal aortas, the amount of positive staining lipid within a determinate area is measured and related to the size of the measured area.

**Quantitative Reverse Transcript Polymerase Chain Reaction**

**Fluorochrome-tagged TaqMan primers**

| All FAM-MGB | Thermofisher 4331182 |
| --- | --- |
| VCAM | Vcam1 Mm01320970_m1 FAM-MGB 250 rxns |
| ICAM | Mm00516023_m1 Icam1 FAM MGB 250 µl |
| CCL-2 (MCP-1) | Mm00441242_m1 CCL2 FAM MGB 75µl |
| CCL-5 | Mm01302427_m1 CCL5 FAM MGB 75 µl |
| CXCL-1 (KC) | Mm04207460_m1 |
| CXCL-2 (MIP-2) | Mm00436450_m1 CXCL2 (MIP-2) FAM-MGB 75 µl |
| IL-4 | Mm00445259_m1 IL-4 FAM-MGB 75 µl |
| IL-6 | Mm00446190_m1 IL-6 FAM-MGB 250 µl |
| IL-10 | Mm00439614_m1 IL-10 FAM-MGB 250 µl |
| TNF-α | Tnf Mm00443258_m1 FAM-MGB 250 rxns |
| TGF-β | Tgfb1 Mm01178820_m1 FAM-MGB 250 rxns |
| IFN-γ | Mm01168134_m1 IFNG FAM-MGB 250 µl |
| IL-1β | Il1b Mm00434228_m1 FAM-MGB 250 rxns |
| NLRP3 | Nlrp3 Mm00840904_m1 FAM-MGB 250 rxns |
| PYCARD/ASC | Mm00445747_g1 PYCARD (ASC) FAM-MGB 250 µl |
| TBX-21 | Mm00450960_m1 TBX21 (T-BET) FAM-MGB 75 µl |
| RORC | Mm01261022_m1 RORC (RORγT) FAM-MGB 75 µl |
| FOXP3 | Mm00475162_m1 FOXP3 FAM-MGB 75 µl |
| GATA3 | Mm00484683_m1 GATA3 FAM-MGB 75 µl |
| P2X7 | Mm01199500_m1 P2rx7 FAM-MGB 250 µl |
| β-Actin VIC | Mm02619580_g1 ACTB (beta-Actin) VIC-MGB Size L (2900 rxns)  Thermofisher 4448491 |

1. Stachon, P., et al., *P2Y6 deficiency limits vascular inflammation and atherosclerosis in mice.* Arterioscler Thromb Vasc Biol, 2014. **34**(10): p. 2237-45.
